# Supplementary figures and images for: Impact of Enhanced Recovery After Surgery (ERAS) protocol versus standard of care on postoperative Acute Kidney Injury (AKI): A meta-analysis
Source: PLoS One. 2021 May 20;16(5):e0251476. doi: 10.1371/journal.pone.0251476 (PMC8136724; doi:10.1371/journal.pone.0251476)

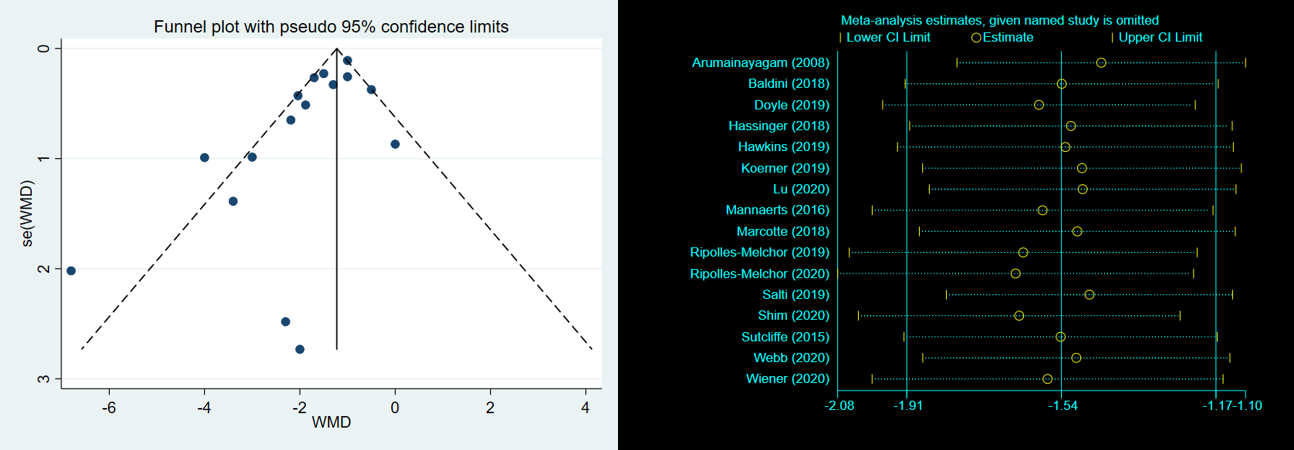


**S2 Fig.** A. Funnel plots for LOS; B. Sensitivity analysis plots for LOS.

Supplement: S2 Fig — A. Funnel plots for LOS; B. Sensitivity analysis plots for LOS. Abbreviations: LOS = length of stay. (DOCX) [file pone.0251476.s002.docx]

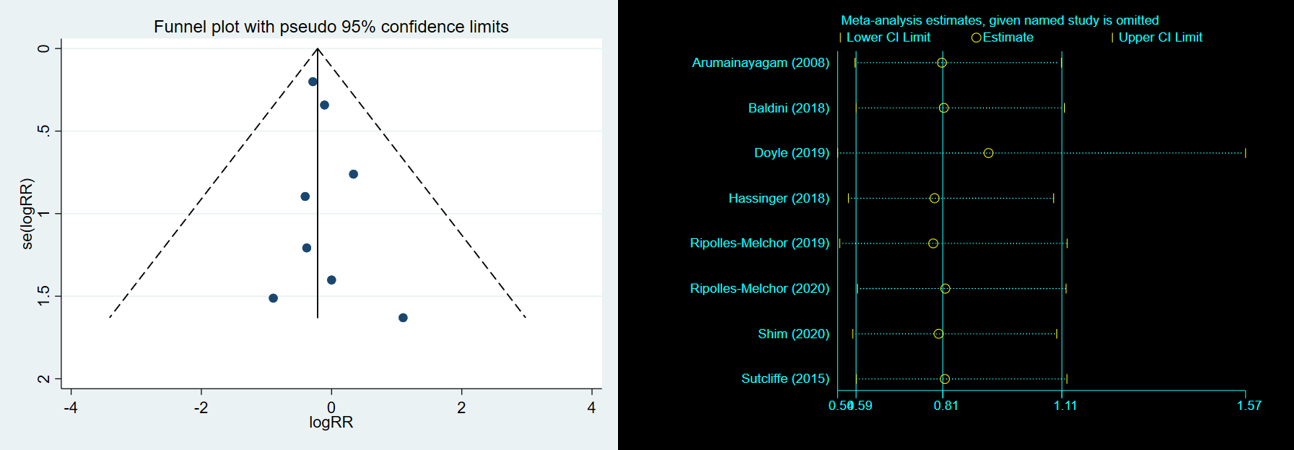


**S5 Fig.** A. Funnel plots for mortality rate; B. Sensitivity analysis plots for mortality rate.

Supplement: S5 Fig — A. Funnel plots for mortality rate; B. Sensitivity analysis plots for mortality rate. (DOCX) [file pone.0251476.s005.docx]
