# Supplementary material for: Impact of Enhanced Recovery After Surgery (ERAS) protocol versus standard of care on postoperative Acute Kidney Injury (AKI): A meta-analysis
Source: PLoS One. 2021 May 20;16(5):e0251476. doi: 10.1371/journal.pone.0251476 (PMC8136724; doi:10.1371/journal.pone.0251476)
Supplement: S3 Fig — A. Funnel plots for 30-day readmission rate; B. Sensitivity analysis plots for 30-day readmission rate. (DOCX) [file pone.0251476.s003.docx]

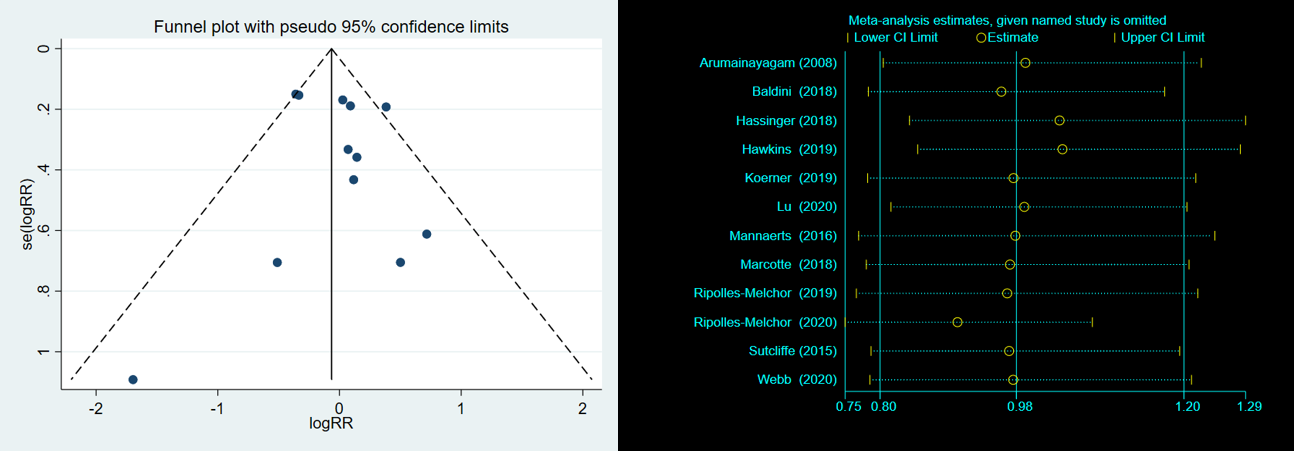


**S3 Fig.** A. Funnel plots for 30-day readmission rate; B. Sensitivity analysis plots for 30-day readmission rate.
