# Supplementary material for: Impact of Enhanced Recovery After Surgery (ERAS) protocol versus standard of care on postoperative Acute Kidney Injury (AKI): A meta-analysis
Source: PLoS One. 2021 May 20;16(5):e0251476. doi: 10.1371/journal.pone.0251476 (PMC8136724; doi:10.1371/journal.pone.0251476)
Supplement: S1 Appendix — (DOCX) [file pone.0251476.s006.docx]

**S1 Appendix. PUBMED search equation**

**Search: ((enhanced recovery after surgery[Title/Abstract]) OR (ERAS[Title/Abstract]) OR (fast-track surgery[Title/Abstract]) OR (FTS[Title/Abstract])) AND ((acute kidney injury) OR (AKI) OR (acute renal failure) OR (ARF) OR (complications) OR (outcomes))**

((("enhanced recovery after surgery"[Title/Abstract] OR "ERAS"[Title/Abstract]) OR "fast track surgery"[Title/Abstract]) OR "FTS"[Title/Abstract]) AND ((((((("acute kidney injury"[MeSH Terms] OR (("acute"[All Fields] AND "kidney"[All Fields]) AND "injury"[All Fields])) OR "acute kidney injury"[All Fields]) OR "AKI"[All Fields]) OR (((("acute kidney injury"[MeSH Terms] OR (("acute"[All Fields] AND "kidney"[All Fields]) AND "injury"[All Fields])) OR "acute kidney injury"[All Fields]) OR (("acute"[All Fields] AND "renal"[All Fields]) AND "failure"[All Fields])) OR "acute renal failure"[All Fields])) OR "ARF"[All Fields]) OR (((((((("complicances"[All Fields] OR "complicate"[All Fields]) OR "complicated"[All Fields]) OR "complicates"[All Fields]) OR "complicating"[All Fields]) OR "complication"[All Fields]) OR "complication s"[All Fields]) OR "complications"[MeSH Subheading]) OR "complications"[All Fields])) OR ("outcome"[All Fields] OR "outcomes"[All Fields]))
