# Supplementary material for: Impact of Enhanced Recovery After Surgery (ERAS) protocol versus standard of care on postoperative Acute Kidney Injury (AKI): A meta-analysis
Source: PLoS One. 2021 May 20;16(5):e0251476. doi: 10.1371/journal.pone.0251476 (PMC8136724; doi:10.1371/journal.pone.0251476)
Supplement: S1 Raw material — (DOCX) [file pone.0251476.s009.docx]

**Raw Data**


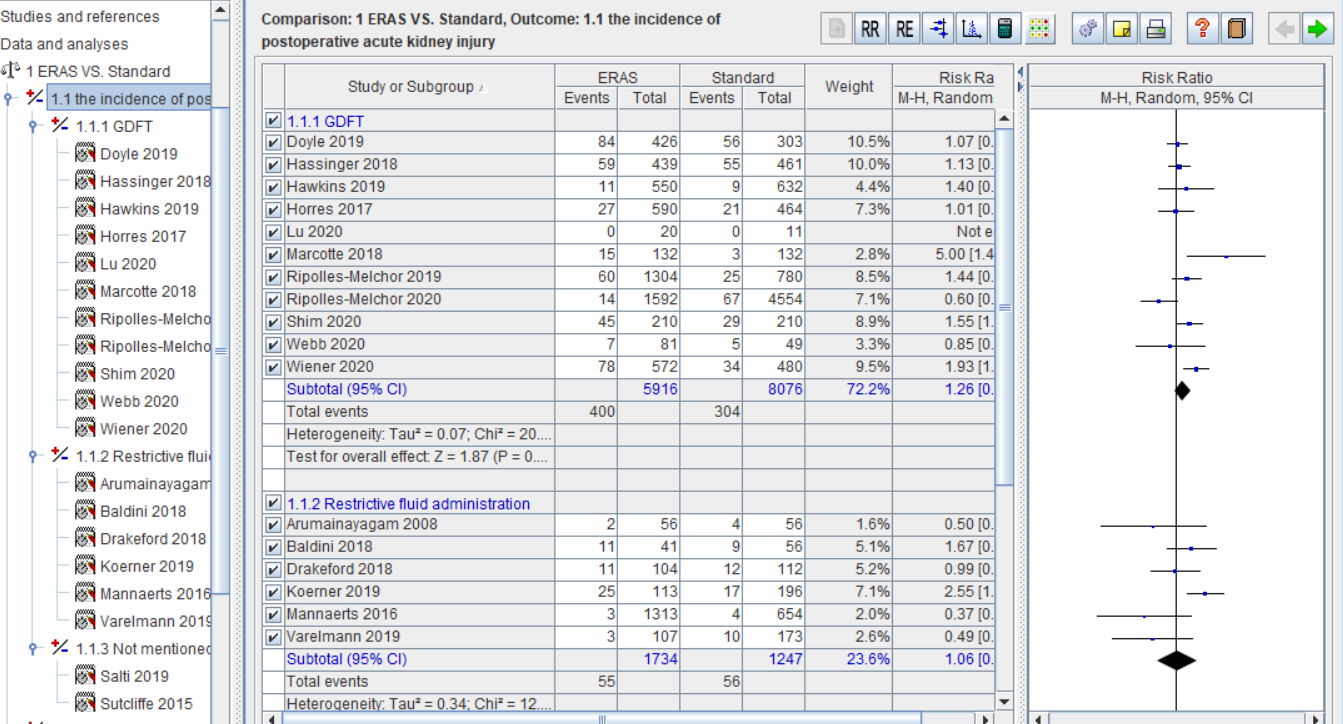


**Fig 1.** Raw data on effect of different fluid management modes on postoperative AKI.


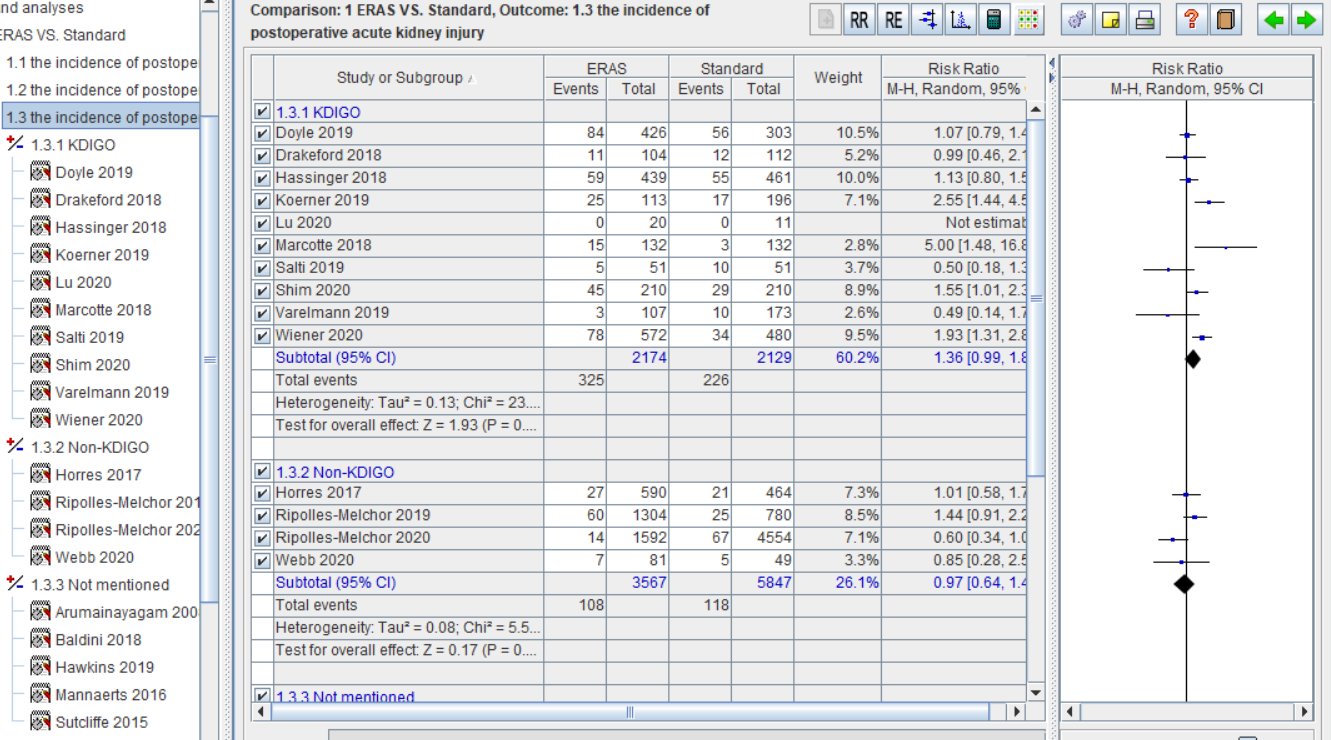


**Fig 2.** Raw data on effect of different diagnostic criteria on postoperative AKI.


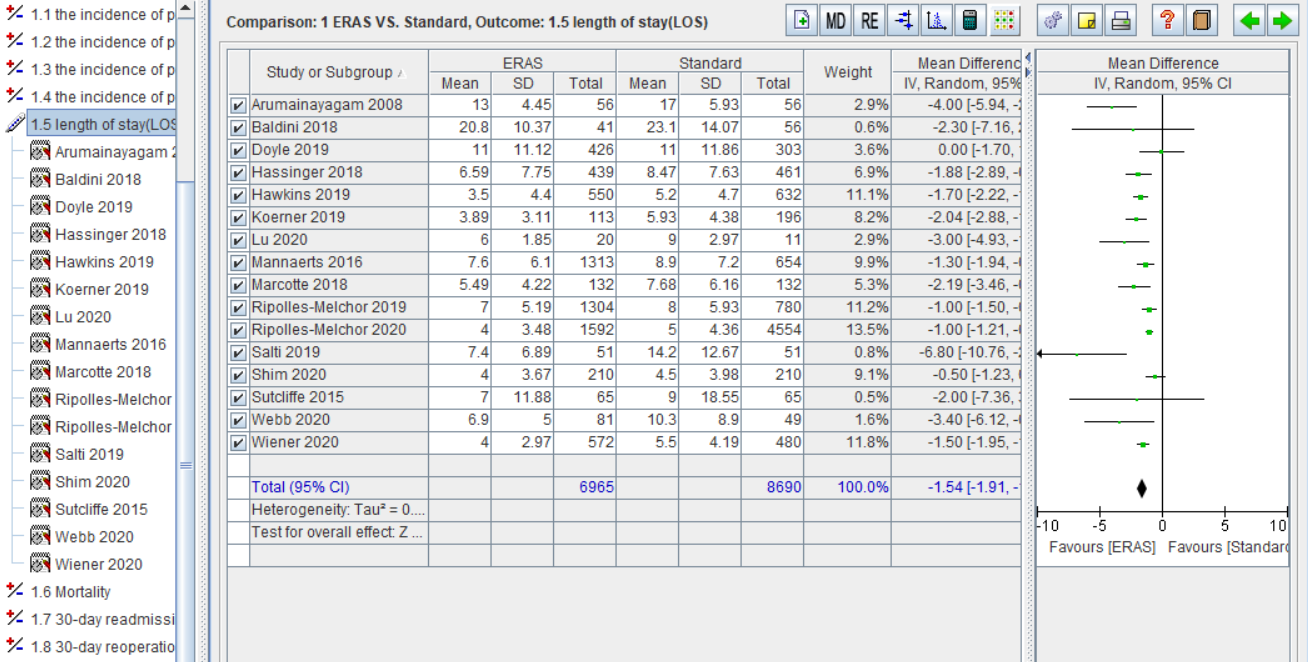


**Fig 3.** Raw data of ERAS protocol effect on length of stay.


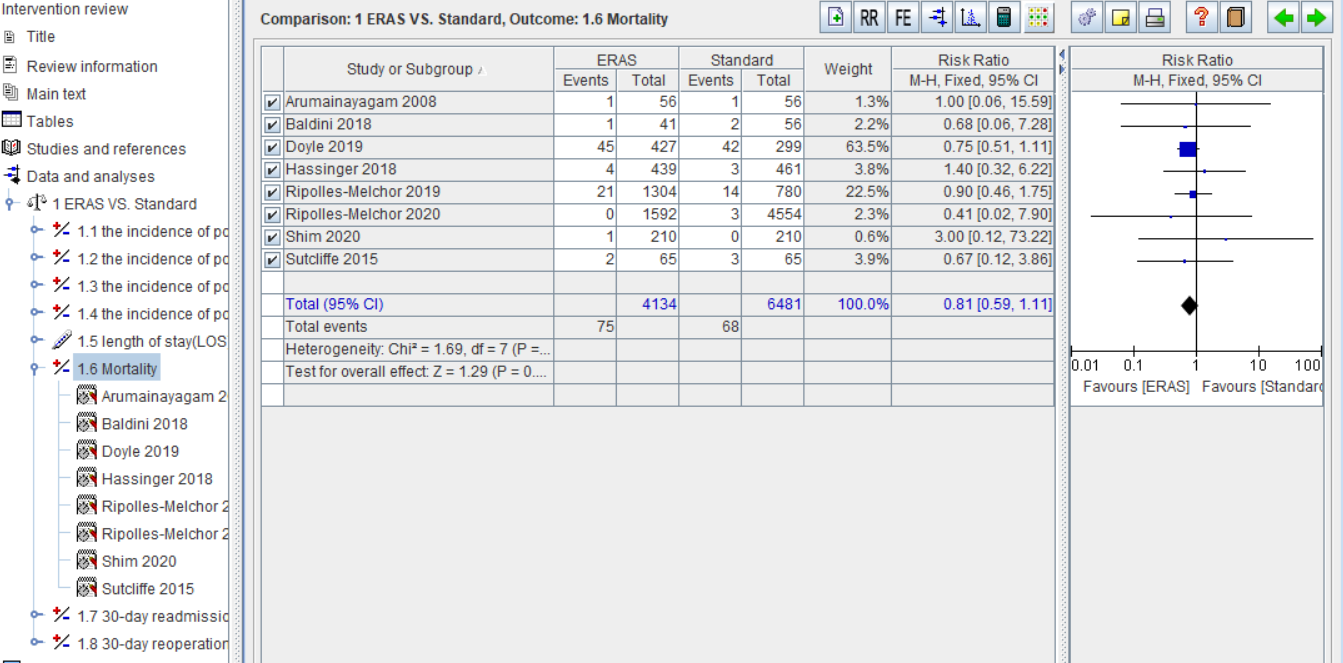


**Fig 4.** Raw data of effect of ERAS protocol on mortality.


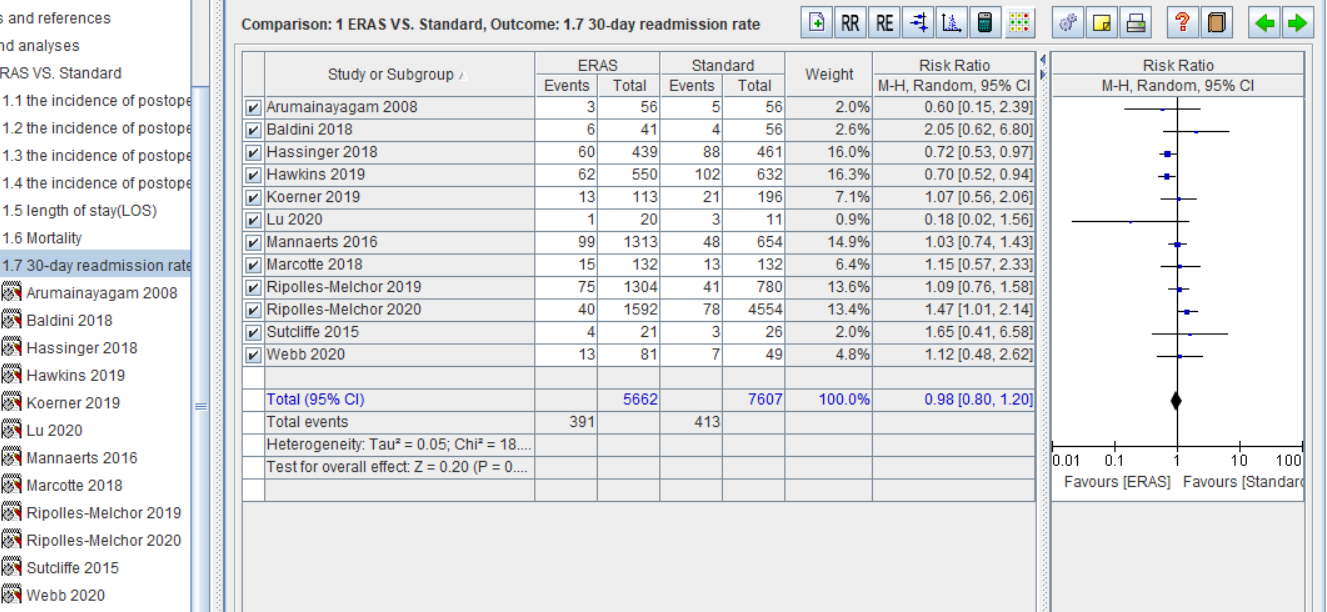


**Fig 5.** Raw data on the effect of ERAS protocol on 30-day readmission rate.


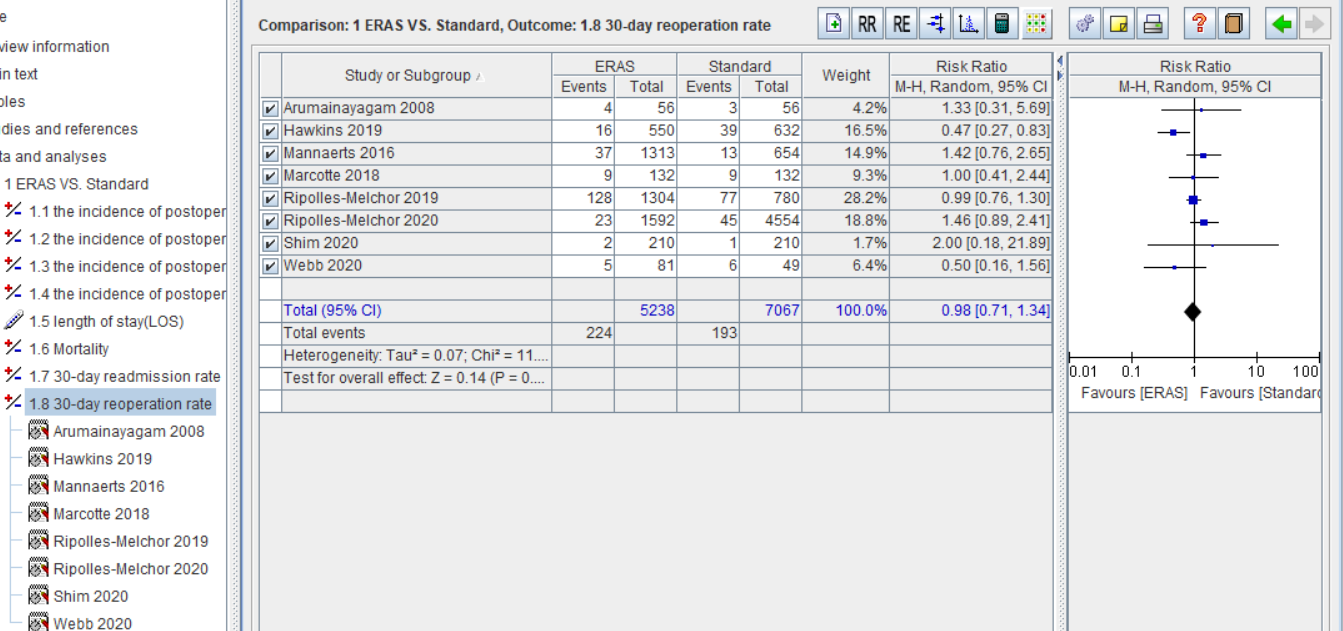


**Fig 6.** Raw data on the effect of ERAS protocol on 30-day reoperation rate.
